# Seasonal Relationship of Prosthetic Joint Infection Following Primary Total Joint Arthroplasty in a Subtropical Climate: A Retrospective Cohort Study

**DOI:** 10.31486/toj.22.0086

**Published:** 2022

**Authors:** Lacey Giambelluca, Brian Godshaw, Jimmy Daher, George Chimento

**Affiliations:** Department of Orthopedic Surgery, Ochsner Clinic Foundation, New Orleans, LA; The University of Queensland Medical School, Ochsner Clinical School, New Orleans, LA

**Keywords:** *Arthroplasty–replacement*, *prosthesis–related infections*, *seasons*, *temperature*, *weather*

## Abstract

**Background:** One devastating complication that leads to increased morbidity and mortality rates after total joint arthroplasty (TJA) is prosthetic joint infection (PJI). Evidence on the relationship between climate, seasonality, and the risk of developing a PJI conflicts. The objective of this study was to investigate the effect of seasonality and climate change on the rate of PJI.

**Methods:** We retrospectively reviewed data of patients undergoing primary TJA at a single institution in a subtropical climate location from 2012 to 2015. Only primary TJAs with a minimum of 1-year follow-up were included in the analysis. Patient demographics and complications were extracted from the database, and monthly average temperature, humidity, and precipitation were obtained. The primary endpoint was PJI requiring revision surgery within 1 year of the index procedure.

**Results:** A total of 3,696 TJAs met the inclusion criteria, with 28 PJIs requiring a second surgery within 1 year (0.76%). We found no significant difference in age, sex, or body mass index in patients who developed a PJI (*P*=0.9450, *P*=0.0989, and *P*=0.7942, respectively). The highest incidence of PJI occurred in August (1.49%), but the incidence of PJI by month was not significant (*P*=0.8996). July and August were the hottest (91 °F) and most humid (79%) months, and June had the most average precipitation (8.06 inches); however, these climate variables were not significant contributors to the incidence of PJI (*P*=0.4996, *P*=0.4999, and *P*=0.4957, respectively).

**Conclusion:** We found no association between temperature, humidity, and development of PJI in a North American subtropical climate. Surgeons can use this information to counsel patients when planning for TJA.

## INTRODUCTION

Total joint arthroplasty (TJA) is an effective surgical option for patients with osteoarthritis and generally results in significant pain relief and improved quality of life.^1^ The demand for TJA has increased during the past several decades, and a 174% increase in total hip arthroplasty (THA) and a 673% increase in total knee arthroplasty (TKA) are projected from 2005 to 2030.^2^ Prosthetic joint infection (PJI), a devastating complication of TJA, is the leading cause for revision surgery after TKA and the third most common reason for revision after THA.^3^ Revision surgeries after PJI either by irrigation and debridement or a 2-stage exchange arthroplasty have been associated with worse outcomes, with a reinfection rate of 25% for the 2-stage exchange arthroplasty and 50% for irrigation and debridement surgeries.^4^ Additionally, PJI results in significant health care expenditure nationwide, with the annual cost to US hospitals projected to exceed $1.85 billion by 2030 for combined hip and knee surgeries ($753.4 million for THA and $1.1 billion for TKA).^5^

Several patient and environmental factors contribute to the development of PJI. Diabetes mellitus, body mass index (BMI), renal failure, peripheral vascular disease, and revision surgery are well established in the literature to increase a patient's risk of developing an infection postoperatively. Additionally, the efficacy of perioperative preventive measures, including bacterial decolonization, prophylactic antibiotics, surgeon scrubbing, proper draping technique, and laminar airflow operating rooms, are environmental factors that can influence patient risk for infection.^3,6,7^

The increase in demand for TJA coupled with the health and economic burden of PJI has peaked interest in identifying additional risk factors for PJI.^7-10^ Haws et al investigated seasonal variations as a possible risk factor for infection.^11^ Current hypotheses for an association include increased bacterial skin colonization in the summer because of higher temperatures and humidity vs increased indoor contact during the winter.^12-14^ Studies have investigated the effect of seasonality on infection rates following various orthopedic surgeries,^15-18^ but the results have been inconsistent. Some studies showed a higher rate of infection during summer,^15,18^ another showed an increased rate during winter,^16^ and the study by Haws et al showed no difference in PJI rate when comparing warm months (May to September) vs cold months (October to April).^11^

Data on specific climate variables contributing to seasonal variations in infection are lacking. The effect of climate-specific data such as temperature, humidity, and precipitation on the incidence of PJI has not been investigated. We conducted this study to investigate the effect of seasonality on the rate of PJI and to examine the impact of specific climate data at the time of primary TJA on the development of PJI.

## METHODS

After approval from our institutional review board, we conducted a retrospective review of prospectively collected data. All patients undergoing TJA from 2012 to 2015 at a single institution in a North American subtropical climate were reviewed. Inclusion criteria included primary THA or TKA and a minimum of 1-year clinical follow-up. Revision procedures were excluded. We collected date of surgery and patient demographics: age, sex, and BMI. American Society of Anesthesiologists (ASA) physical status classification was collected for all patients and used as an indicator of preoperative comorbidities. For the purposes of this study, PJI was defined according to the Musculoskeletal Infection Society criteria and as patients requiring return to the operating room for the following procedures: arthrotomy, including irrigation and debridement; polyethylene exchange; or 1- or 2-stage revision procedures.^19^ Superficial complications such as seromas, stitch abscesses, and cellulitis were not considered PJI in this study. The primary endpoint was PJI requiring revision surgery within 1 year of the index procedure.

All patients underwent a standardized perioperative routine. Anesthesia was administered by spinal epidural. All patients received perioperative antibiotics following a standardized regimen of vancomycin and cefazolin preoperatively and 2 doses of cefazolin postoperatively. If patients had a documented severe allergic reaction to penicillin, clindamycin was substituted for cefazolin. Postoperative thromboembolic prophylaxis followed a standardized regimen of warfarin or aspirin, based on current literature at the time of the index procedure.

Climate data were obtained from publicly available online databases (Weather Atlas, weather.us, and The Weather Channel). Data obtained included monthly average high temperatures and low temperatures, humidity, and average precipitation in inches. For all PJIs, the high and low temperatures were obtained from the date of the index procedure. Index procedures were grouped by the season in which they were performed: winter (December, January, and February), spring (March, April, and May), summer (June, July, and August), and fall (September, October, and November). These months were chosen for each season, rather than exact dates, to allow for collection of monthly average temperatures.

Data were collected using Excel (Microsoft Corporation). Tests were performed with a significance level of α=0.05, and any values were considered statistically significant if *P*<α. Chi-square test and Fisher exact test were used as appropriate for month, season, temperature, humidity, precipitation, ASA physical status classification, and PJI. Correlation coefficient, R^2^, is reported for the correlation between PJI incidence rate and temperature, humidity, precipitation, and month as continuous variables. A power analysis with an α cutoff of 5% and β cutoff of 20% was used to determine the minimum number of subjects needed to be enrolled to have sufficient statistical power to detect a PJI. A minimum of 1,592 subjects was needed for an adequate study power.

## RESULTS

A total of 3,696 primary TJAs (1,070 THAs and 2,626 TKAs) performed by 5 fellowship-trained orthopedic surgeons were included in this study. Twenty-eight PJIs (0.76%) were identified: 12 THAs (42.9%) and 16 TKAs (57.1%). The incidence of PJI was similar for both THA and TKA, with an incidence of 1.1% within 1 year of THA and 0.6% of TKA. We found no significant differences in age (*P*=0.9450), sex (*P*=0.0989), or BMI (*P*=0.7942) in patients who developed a PJI (Table 1).

**Table 1.** Patient Demographics

Variable	All Patients, n=3,696	Prosthetic Joint Infection, n=28	*P* Value
Surgery			
Total hip arthroplasty	1,070 (29.0)	12 (42.9)	0.1568
Total knee arthroplasty	2,626 (71.0)	16 (57.1)	
Sex			
Male	1,372 (37.1)	15 (53.6)	0.0989
Female	2,324 (62.9)	13 (46.4)	
Age, years, mean	64.8	66.0	0.9450
Body mass index, kg/m^2^, mean	32.8	32.5	0.7942

Note: Data are presented as n (%) unless otherwise indicated.

**Figure 1.**
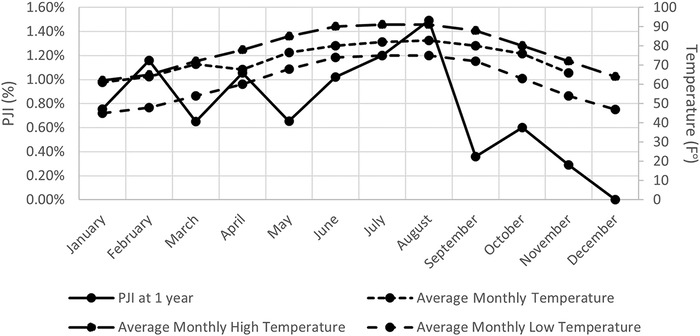
Prosthetic joint infection (PJI) and temperature by month. No correlation was found between PJI and average monthly high temperature, average monthly low temperature, or average monthly temperature at index surgery.

Thirteen organisms were isolated from cultures, and 8 patients (28.6%) had no growth on their intraoperative cultures (Table 2). The highest incidence of PJI was in August (1.49%); however, the comparison of the number of PJIs per month showed no effect of month on PJI and was not statistically significant (*P*=0.8996). As shown in Table 2, we found no significant difference in infection rates between months based on temperature, humidity, and precipitation (*P*=0.5). Figure 1 shows the average temperatures per month and their correlations with PJI; no correlation was detected between PJI and average monthly high temperature, average monthly low temperature, or average monthly temperature at index surgery.

**Table 2.** Prosthetic Joint Infection Incidence, Climate Variables, and Cultures by Month From 2012 to 2015

Month	TJA, n	PJI, n (%)	Average High Temperature, °F	Average Low Temperature, °F	Average Humidity, %	Average Precipitation, Inches	Cultures
January	398	3 (0.75)	62	45	76	5.15	MSSA (2) MRSA
February	345	4 (1.16)	65	48	73	5.46	*Gemella morbillorum* *Staphylococcus epidermidis* NG (2)
March	307	2 (0.65)	72	54	73	4.55	*Candida albicans* Skin flora
April	284	3 (1.06)	78	60	73	4.61	*Enterococcus faecalis* MSSA *Serratia marcescens*
May	306	2 (0.65)	85	68	73	4.63	*Corynebacterium jeikeium* MRSA
June	294	3 (1.02)	90	74	76	8.06	*Enterococcus faecalis* NG (2)
July	250	3 (1.20)	91	75	79	5.93	GBS *Klebsiella pneumoniae*-ESBL MSSA
August	268	4 (1.49)	91	75	79	5.98	*Streptococcus dysgalactiae* NG (3)
September	278	1 (0.36)	88	72	78	5.05	MSSA
October	334	2 (0.60)	80	63	75	3.58	MAC *Serratia marcescens*
November	345	1 (0.29)	72	54	77	4.49	NG
December	287	0 (0.00)	64	47	77	5.33	–
PJI vs climate variable, *P* value			0.5	0.5	0.5	0.5	

ESBL, extended spectrum beta-lactamase; GBS, group B streptococcus; MAC, *Mycobacterium avium* complex; MRSA, methicillin-resistant *Staphylococcus aureus*; MSSA, methicillin-susceptible *Staphylococcus aureus*; NG, no growth; PJI, prosthetic joint infection; TJA, total joint arthroplasty.

**Figure 2.**
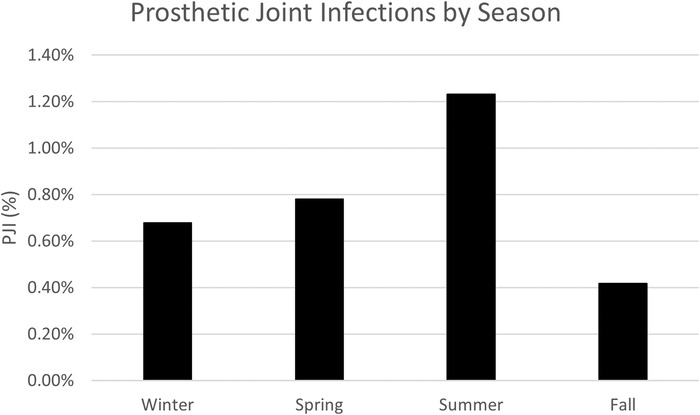
The highest percentage of prosthetic joint infection (PJI) occurred during the summer (1.23%) and the lowest incidence occurred in the fall (0.42%), but the difference between seasons was not significant (*P* value=0.9985).

July and August were the hottest (91 °F) and most humid (79%) months, and June had the most average precipitation (8.06 inches); however, these climate variables were not significant contributors to the incidence of PJI (*P*=0.4996, *P*=0.4999, and *P*=0.4957, respectively) (Table 3). No PJIs occurred in patients whose index procedure was performed in December, and we have no relevant findings in our data to explain the significance of this finding. As shown in Table 3, the incidence of PJI poorly correlated with average high temperature (R^2^=0.42717), average low temperature (R^2^=0.43205), average precipitation (R^2^=0.42527), and average humidity (R^2^=0.021098). When stratified by season, we found no significant difference in the incidence of PJI (*P*=0.9985, Figure 2, Table 4). Overall, we found no significant relationship between the incidence of PJI and average temperature, precipitation, humidity, or month at the time of primary TJA. Finally, the relationship between ASA physical status classification and PJI risk was not statistically significant (*P*=0.13, Table 5).

**Table 3.** Correlation of Prosthetic Joint Infection (PJI) Incidence to Climate Variables and Month From 2012 to 2015

Comparison	Correlation Coefficient, R^2^	*P* Value
PJI vs average high temperature	0.42717	0.4996
PJI vs average low temperature	0.43205	0.4996
PJI vs average temperature	0.25611	0.4997
PJI vs average precipitation	0.42527	0.4957
PJI vs average humidity	0.021098	0.4999
PJI vs month	–0.50414	0.5016

**Table 4.** Prosthetic Joint Infection (PJI) Incidence by Season From 2012 to 2015

Season	Total Surgeries, n	PJI by 1 Year, n	PJI Incidence, %	*P* Value
Winter	1,030	7	0.68	0.9985
Spring	897	7	0.78	
Summer	812	10	1.23	
Fall	957	4	0.42	

**Table 5.** Prosthetic Joint Infection (PJI) Incidence by American Society of Anesthesiologists (ASA) Physical Status Classification

ASA Physical Status Classification	Total Surgeries, n	Noninfectious, n (%)	PJI, n (%)	*P* Value
1	57	57 (100)	0 (0.0)	0.13
2	1,805	1,796 (99.5)	9 (0.5)	
3	1,800	1,782 (99.0)	18 (1.0)	
4	34	33 (97.1)	1 (2.9)	

## DISCUSSION

Given the patient morbidity and mortality, coupled with the significant health care burden of PJI, identifying risk factors and developing preventive measures to reduce the risk of postoperative complications after TJA are imperative. This study sought to determine the effect of climate, a nonmodifiable risk factor, on the incidence of PJI following TJA. To our knowledge, this study provides the most detailed weather data in an investigation of whether seasonal climate variations affect PJI incidence. In this retrospective study, we found no significant relationship between the season, month, average temperature, humidity, or precipitation when a primary TJA was performed and the subsequent development of a PJI. We found an overall incidence of 0.76% PJI requiring a revision surgery within 1 year, an incidence that is in line with previous studies.^10,20,21^

While most studies have found that seasonal variations had a significant effect on PJI in THA,^15-18^ we were unable to corroborate. Our results were more consistent with findings of Haws et al.^11^ This difference in results may be attributable to multiple factors. A Medicare database analysis of more than 1 million patients found that season had a significant effect on PJI of THA in the southern region of the United States.^16^ Rosas et al noted that the southern region had the highest incidence of PJIs in the winter (1.1%), which was significantly higher than the incidence rate in the fall (0.96%, *P*<0.05) but not significantly higher than in any other season. Rosas et al stated that a database analysis allows for a greater sample size but potentially introduces reporting bias and confounders, including differences in perioperative prophylaxis protocols and patient-specific risk factors.^16^ Our patients, on the other hand, were on a standardized protocol for thromboembolic prophylaxis and perioperative antibiotics.

Multiple studies have shown infections caused by different types of bacteria such as *Staphylococcus aureus* peak in the summer months,^13,22,23^ hypothesized to be attributable to increased skin colonization secondary to the higher temperature and humidity, which is supported by multiple laboratory and clinical studies.^24,25^ Kane et al conducted a retrospective analysis of 17 infections following TJA at a single institution.^18^ The infection incidence was the highest during summer (4.7%), decreasing to 2.4% in the fall and 1.5% in the winter, and reaching the lowest rate of infection during the spring (0.5%), a gradual decrease in infection rate from summer to spring. Further, when comparing months, August had the highest PJI incidence rate of 5.4%, followed by July (4.5%) and September (4.3%), and the comparison of the summer/fall months vs winter/spring months was statistically significant (*P*=0.013).^18^ We found that July and August were the hottest and most humid months, and August had the highest incidence of PJI, but the correlation with climate variables was not significant.

This study has limitations. First, the retrospective design of this study may have resulted in some patients being lost to follow-up. Second, this study did not account for specific patient comorbidities; instead, we used ASA physical status classification as an indicator of preoperative risk. However, because the purpose of this study was solely to investigate seasonal climate variations and their effects on PJI for all patients undergoing TJA, investigating specific comorbidities would have been out of the scope of this study. Another possible limitation is accounting for the *July effect* when new and inexperienced surgical residents are involved in TJAs, and higher rates of surgical infections may occur during their first few months in training.^26,27^ Gruskay et al published a review of more than 8,100 spine surgeries at a single tertiary referral institution and noted a significantly higher rate of surgical site infection (SSI) during the summer months.^28^ The authors were unable to determine if the higher SSI rate observed during the summer was related to house staff experience or seasonal variation.^28^ Even if the July effect is associated with increased risks of infection, we did not see it in this study. Finally, our study evaluates PJI within 1 year of index surgery, so further studies with long-term follow-up are warranted.

## CONCLUSION

PJIs are a challenging complication of TJA, so identifying risk factors for the development of PJI is critical to ensure successful outcomes. Surgeons should reassure patients when planning a primary TJA that temperature, climate, season, and humidity were not shown in our study to increase the risk of PJI in a subtropical climate. Optimization of modifiable patient risk factors remains one of the most effective ways to reduce the risk of developing a PJI.

## Notes

*This article meets the Accreditation Council for Graduate Medical Education and the American Board of Medical Specialties Maintenance of Certification competencies for Patient Care, Medical Knowledge, and Practice-Based Learning and Improvement.*
